# Supplementary material for: Patient-level predictors of diabetes-related lower extremity amputations at a quaternary hospital in South Africa
Source: PLoS One. 2020 Oct 16;15(10):e0240588. doi: 10.1371/journal.pone.0240588 (PMC7567354; doi:10.1371/journal.pone.0240588)
Supplement: Attachment — Submitted filename: Response to reviewers.docx [file pone.0240588.s002.docx]

Manal S. Fawzy, Ph.D., M.D.
Academic Editor
PLOS ONE

Dear Sir

Thank you for providing us an opportunity to revise the manuscript- PONE-D-20-15807. Please find a response to all points of the review.

Kind Regards

Dr ST Mtshali

16August 2010

General Findings

1. Please ensure that your manuscript meets PLOS ONE's style requirements,

We have revised the Title page and main document to conform to the style requirement of the journal

2. In the ethics statement in the manuscript and in the online submission form, please provide additional information about the patient records used in your retrospective study. Specifically, please ensure that you have discussed whether all data were fully anonymized before you accessed them and/or whether the IRB or ethics committee waived the requirement for informed consent. If patients provided informed written consent to have data from their medical records used in research, please include this information.

The following statement has been added on line 102 and 103 of the revised manuscript- Data were retrieved from an electronic database, with no patient level identifiers. The ethics committee waivered requirements for Informed consent

3. Please address the following:
   - Please describe your study variables in further detail within your Methods section.
   - Please state the date(s) upon which these medical records were accessed.

Line 95-97: The following variables were retrieved from the electronic database: Sociodemographic variables including Age, gender, race, type of diabetes, hospital status, additional diabetes related medical complications and lower extremity amputation (new or present).

Comments:
1. The introduction starts by saying “Diabetes account for most morbidity and mortality globally”. I am not sure that is really accurate – if it is – then a reference to the statement would be important. Its certainly an important contributor, but not sure if it accounts for most morbidity and mortality.

The sentence has been modified to reflect that Diabetes is an important contributor morbidity and mortality globally

2. The objective at one point says “incidence” and at one point says “prevalence” for lower extremity amputation – these are not interchangeable.

We have clarified this to reflect that the aim of the study was to determine the **overall prevalence** of LEA and associated risk factors for LEA amongst diabetes patients that were referred to the Central hospital (IALCH) between 1 January 2014 and 31 December 2015. We also disaggregated the number of amputations performed at the IALCH

3. The definition of the cohort with diabetes should be better described in the methods. The data collection section say that data were retrieved “for each diabetes patient that was managed during the study period”. Does this mean people with a known diagnosis of diabetes at admission? People who met a certain blood glucose or HbA1c criterion?

Patients were classified with a diagnosis of Diabetes based on the ICD 10 coding allocated to the patient between E 10.1 and E14.9

4. How was data on the outcome of lower extremity amputation obtained from the electronic record – was this done by billing code for the surgery? By discharge diagnosis? A better definition of the study outcome would be helpful. Similarly, how was “type of diabetes” ascertained and defined?

The diagnosis were based on ICD 10 code allocated to the patient. The CPT 4 procedure code for amputation was used to determine patients with an amputation

5. The authors note that 99 of 234 amputations were new amputations conducted at the quaternary care facility. How were amputations performed elsewhere ascertained?

Over these two years, 234 (17 per 1000) of the diabetes patient attending IALCH had an ICD_ 10 code related to amputations. Ninety-nine (7 per 1000) of the diabetes patients attending IALCH were coded as new amputations conducted at IALCH, accounting for 2% of all diabetes admissions.

6. It is confusing that the paper seems to be all about hospitalized patients, but there is a row for “never admitted” in the table. Is it that people weren’t admitted for their amputation? This needs to be clarified.

The never admitted were referring to their outpatients consultation and tehse patients were not inpatients for amputation. In order to avoid the confusion- we have deleted these two rows from the table

7. Were all amputations or only those classified as “new” included in the models?

We inputted both in the model but have now removed the all amputations column as they do not directly relate to this paper.

8. A major limitation is lack of measures of diabetes control (HbA1c) as a predictor. This should be mentioned in the limitations section.

Thank you for this input. We have added this to limitations: Information related to the patients diabetic control (Hb1AC levels) were not collected and poor diabetes control could have been a significant contributor to adverse outcomes including amputations.

9. In the conclusion, would temper the statement in the first sentence with – “In our study of patients with diabetes admitted to a quaternary care facility in South Africa, patients with NIDDM and who were female…..”

Thank you. This information was modified.

10. The authors mention HIV in the second to last sentence. The interaction between HIV and diabetes is a complex one. Without information on HIV status and ART regimen, I am not sure anything can be concluded about that interaction from this paper, although I do agree that this interaction is important. Perhaps they can make the lack of data on HIV status and ART a study limitation, which would make this concluding statement about HIV have some additional context.

Noted and added to the study limitations

Reviewer #2: In their manuscript “Patient-level predictors of diabetes-related lower extremity amputations at a quaternary hospital in South Africa”, the authors describe 234 patients who underwent amputation among 13,742 patients with diabetes.
1. Were these toe amputations? Above-knee amputations? Below-knee amputations? Did any patient have more than one amputation? These data points need to be determined.

Data on amputations were collected for lower extremity amputations and include both above and below knee amputations. This was based on ICD 10 diagnosis. We did not disaggregate the data as we were concerned with overall amputation status. We counted the incidence per patient once only irrespective of the number of amputations.

2. It is unclear what the difference is in Table 2 between the crude analyses and the adjusted analyses.

We have modified the table
3. The discussion initially describes a rate of 234 amputations out of 13742 patients (1.70%) as 1.73%, and then for some reason changes the numbers to 99 out of the same denominator. Next, several other regions are quoted for context, but it is not clear to the reader why these regions are chosen. Overall this is confusing.

This has been corrected in the manuscript

4. The conclusions state that better screening is needed, but this is incidence data. What does that have to do with the need for better screening?

This statement has been modified: Specific guidelines should be developed for primary health care practitioners on the frequency, clinical and laboratory tests required for early diagnosis and referrals for patients with poor diabetes control and early signs of diabetes-related complications
