# Supplementary material for: Patient-level predictors of diabetes-related lower extremity amputations at a quaternary hospital in South Africa
Source: PLoS One. 2020 Oct 16;15(10):e0240588. doi: 10.1371/journal.pone.0240588 (PMC7567354; doi:10.1371/journal.pone.0240588)
Supplement: Attachment — Submitted filename: Response to reviewers september 2020.docx [file pone.0240588.s003.docx]

Manal S. Fawzy, Ph.D., M.D.
Academic Editor
PLOS ONE

Dear Sir

Thank you for providing us an opportunity to revise the manuscript- PONE-D-20-15807. Please find a response to all points of the review.

Kind Regards

S T Mtshali

21 September 2020

Reviewer #1: 1. I found the first sentence of the abstract confusing, and suggest deleting it.

Thank you- Sentence has been deleted
2. The authors note that the purpose of the study is to estimate prevalence, but the title contains with word “incidence” – again these are not interchangeable.

It was an omission on our part. Line 87 page 4 we have changed to incidence as we were concerned with new cases
3. The authors note in response to R2 that all amputations were included (above and below knee) – this should be specified in the manuscript as well.

We have added above and below knee at line 88 on the purpose and line 107
4. Some of the new text in Data Collection needs a bit of editing/spell checking, but appreciate the addition of ICD10 and CPT codes to the methods

We have corrected the spelling between line 105 and 108
